# Criminal Abortion

**Published:** 1878-06

**Authors:** 


Criminal Abortion.—The destruction of human life wantonly has been looked upon in all ages and by all nations and races of people as a grievous crime. Vitality is given the ovum by impregnation, and separate, distinct life from that of the mother, in a new being, commences with conception. Arbitrary and wilful destruction at certain periods of human life thus begun, is marked by different degrees of moral and legal turpitude, according to the age of the new being. Distinct terms are also applied to the killing at these various stages of life. Destruction of life at any time from conception to the full period of utero-gestation is in legal medicine called abortion. At the time of birth and for a few days after, it is called infanticide; and at all subsequent periods of life the term murder is applied. In obstetrics the term abortion, strictly speaking, is the discharge of the ovum before the middle of the fourth month, or time of quickening. After this, to the latter part of the sixth month, or period of viability, the discharge is called miscarriage; and premature delivery from this time to the full term of pregnancy. Conventionalities in certain circles of society fix a standard of moral rules to suit their convenience and pleasure. One of these is that there is no crime in destroying the ovum before the fifth month of pregnancy. This rule is predicated upon the cherished idea that life does not exist till the period of quickening. The word *abortion* does not appear in the technicalities of these moralists, but "produce the menses" and "prevent conception" are the usual terms by which their wishes are made known. Sometimes pregnant females, preferring the pleasures of social society to the duties of domestic life, and apparently ignorant of their condition, apply to physicians for a prescription to restore suppressed menstruation. This is done under the prevailing opinion that whatever will restore suppressed menses will produce abortion—knowing that honorable physicians will not consent to the gratification of their real desire. This plan is so *thin*, and has so often failed to accomplish the object, that it is now seldom resorted to. The rule of the day is to "*prevent conception;*" and the sympathy of physicians is being inlisted for the feeble female, the over-prolific female, the female who suffered so severely at her last confinement and the dashing female who cannot bear the idea of burying herself to the festivities of social society. The wife and the husband implore the doctor for means to prevent conception. A practitioner is touched by the appeal, and casts around for a plan by which he can conscientiously accomplish their desire, without moral impropriety or physical injury to the parties, but all are objectionable. His conscientious duty as a responsible and honorable member of society and of a noble profession compels him to say, let nature take her course—the fiat has been announced, "in pain shalt thou bring forth." Another, less scrupulous, and more inclined to please and receive patronage, can see no impropriety in *preventing life.* In the selection of a plan he is not inclined to adopt some of the more disgusting practices advised, and finally concludes to give ergot before each menstrual period. He manages to satisfy himself and his patient that no wrong can be made of this—that no cessation has yet occurred, nor other evidence of pregnancy, and if, by giving the remedy, all signs should be prevented, then there can be no palpable violation.

This discussion must, however, be confined to the doctor and his patient. The subject will not bear medical criticism. When describing the result of his practice to another physician, he must necessarily confess that, like the man who shot in a way to kill if the object was a deer, and miss if it was a calf, he intended to kill the child if it had been formed, and miss doing anything at all if conception had not taken place. The plan amounts to a kind of trapsetting to catch and kill the new being if it comes in the way. The old idea of dating the commencement of human life at the period of quickening has given way to enlightened views on this subject, and however desirous parents may be to destroy their own offspring, outspoken public opinion makes them hesitate to commit the foul deed after unmistakable evidences of pregnancy are known to others.

Arbitrary, criminal abortion is the crying sin of this country, and is one of the most atrocious evils to which our people are addicted. It commenced long ago in all grades of American society. The slave of Mississippi and the lady of Massachusetts had their abortiva, to which they resorted, when threatened with cares they were unwilling to assume. Those not versed in the use of appropriate remedies, and had the means to do so, employed professional secret abortionists, whom the demand had created out of low quacks and dishonorable members of the medical profession. In the practice thus instituted, the destruction of life and health does not stop with the intended victim. Hundreds of mothers have fallen under the poison used against their children; and often when life is spared, permanent ill health has been the result. Twenty years ago the Southern slave-holder, the Northern clergy, the statesman and morality were shocked at the progress of this evil. Abortionists were prosecuted, ladies censured, and strict watch kept over the female slave. Notwithstanding this, under various phases, the crime of abortion has continued, and invades every grade of society.

Professional abortionists publish ingenious advertisements, in which remedies, ostensibly, for the cure of various disorders are proposed, but with the significant caution that *pregnant females should not take them.* Nostrum emmenagogues are also offered the public, and lest the abortive effect of such preparation may not be generally understood, it is indirectly made known by a caution to pregnant females against their use.

In this war of Americans against their progeny, we had hoped that honorable members of the medical profession would not do worse than occupy neutral ground. It is certainly bad enough to witness unmoved this embryotic slaughter, but when in any way we allow ourselves to give aid and comfort to the enemies of new beings, we become lost to humanity, and prostitute a noble calling to the encouragement of legal, moral and religious crime.

It is generally understood that the profession of medicine has adopted a high moral standard on this subject, and whatever a respectable member sanctions the public are likely to consider unobjectionable. Hence the importance of maintaining a high moral standard on this subject by contemptuous refusal to aid in such diabolical schemes, and by holding up to professional indignation a member who persists in this course.

---

At the annual meeting of the American Microscopical Society of the City of New York, held Tuesday evening, March 26th, 1878, the following officers were elected for the ensuing year: President, John B. Rich, M.D., 1 West 38th Street, N. Y.; Vice-President, Wm. H. Atkinson, M.D., 41 East 9th Street, N. Y.; Secretary, O. G. Mason, Bellevue Hospital, N. Y.; Treasurer, T. d'Oremieulx, 7 Winthrop Place, N. Y.; Curator, John Frey, Bellevue Hospital, N. Y.

---

American Medical Association.—At the recent meeting of the American Medical Association, held at Buffalo, N. Y., the following officers were elected for the ensuing year: Theophilus Parvin, President; W. F. Westmoreland, A. J. Fuller, John Morris and John H. Murphy, Vice-Presidents.

The Association decided unanimously to hold its next annual meeting in Atlanta, Ga., beginning on the first Monday in May next.

The honor conferred upon Atlanta and her distinguished citizen, Dr. W. F. Westmoreland, is highly appreciated, and she will be glad to welcome the Association to her midst.

